# Uncovering pharmacological mechanisms of Wu-tou decoction acting on rheumatoid arthritis through systems approaches: drug-target prediction, network analysis and experimental validation

**DOI:** 10.1038/srep09463

**Published:** 2015-03-30

**Authors:** Yanqiong Zhang, Ming Bai, Bo Zhang, Chunfang Liu, Qiuyan Guo, Yanqun Sun, Danhua Wang, Chao Wang, Yini Jiang, Na Lin, Shao Li

**Affiliations:** 1 Institute of Chinese Materia Medica, China Academy of Chinese Medical Sciences, Beijing 100700, China; 2 MOE Key Laboratory of Bioinformatics and Bioinformatics Division, TNLIST, Department of Automation, Tsinghua University, Beijing 100084, China; 3 Tianjin International Joint Academy of Biotechnology & Medicine, Tianjin 300457, China

## Abstract

Wu-tou decoction (WTD) has been extensively used for the treatment of rheumatoid arthritis (RA). Due to lack of appropriate methods, pharmacological mechanisms of WTD acting on RA have not been fully elucidated. In this study, a list of putative targets for compositive compounds containing in WTD were predicted by drugCIPHER-CS. Then, the interaction network of the putative targets of WTD and known RA-related targets was constructed and hub nodes were identified. After constructing the interaction network of hubs, four topological features of each hub, including degree, node betweenness, closeness and k-coreness, were calculated and 79 major hubs were identified as candidate targets of WTD, which were implicated into the imbalance of the nervous, endocrine and immune (NEI) systems, leading to the main pathological changes during the RA progression. Further experimental validation also demonstrated the preventive effects of WTD on inflammation and joint destruction in collagen-induced arthritis (CIA) rats and its regulatory effects on candidate targets both *in vitro* and *in vivo* systems. In conclusion, we performed an integrative analysis to offer the convincing evidence that WTD may attenuate RA partially by restoring the balance of NEI system and subsequently reversing the pathological events during RA progression.

Rheumatoid arthritis (RA), as a systemic autoimmune disease, is principally characterized by the presence of inflammatory synovitis, the predominance of pro-erosive mediators, and the progressive destruction of cartilage and bone^1^. Since the pathogenesis of RA has not been fully elucidated, the current therapeutic agents such as nonsteroidal anti-inflammatory drugs (NSAIDs), disease modifying antirheumatic drugs (DMARDs), glucocorticoids, and biological response modifiers, which have been used to reduce inflammation, relieve pain, suppress disease activity, prevent joint damage, and slow disease progression, only maintain the patient's quality of life and ability to function, but not cure the disease^2^. Moreover, these agents are still limited by several well-characterized clinical side effects, such as hepatotoxicity, cardiotoxicity and gastrointestinal effects^3^. As a crucial part of complementary and alternative medical systems, Traditional Chinese Medicine (TCM) has been extensively used in the treatment of arthritic diseases for centuries. On the concept of TCM, RA is categorized as "arthromyodynia" (Bi Zheng, Bi syndrome or blockage syndrome)^4^. Various TCM-based herbal formulas and the extracts of herbs have been demonstrated to be effective for relieving the severity of RA. However, the worldwide clinical application of TCM has been hindered by the lack of scientific understanding on its actions. In order to improve their extensive use and enhance their therapeutic effects, it is extremely necessary to explore the scientific basis and the underlying mechanisms of TCM.

Wu-tou decoction (WTD), as a classic TCM formula, was originally recorded in “Jin Kui Yao Lve” written by Chinese medical sage Zhang Zhongjing. It is prepared from a basic formula of five Chinese herbs, including *Radix Aconiti* (Wu Tou), *Herba Ephedrae* (Ma Huang), *Radix Astragali* (Huang Qi), *Raidix Paeoniae Alba* (Bai Shao) and *Radix Glycytthizae* (Gan Cao), and widely produced in China in accordance with the China Pharmacopoeia standard of quality control. In clinical practice, WTD has been extensively used for the treatment of rheumatic arthritis, RA, constitutional hypotension and hemicrania^5^. According to the TCM theory, multiple agents contained in one formula must work synergistically. With regard to WTD, *Radix Aconiti* is the primary component and is believed to be effective in treating rheumatic arthritis and RA; *Herba Ephedrae* serves as the ministerial component to intensify the analgesic function of *Radix Aconiti*; *Radix Astragali* acts as the adjunctive component to invigorate *qi* (vital energy), strengthen the body and reinforce the effect of *Radix Aconiti* and *Herba Ephedrae*; *Raidix Paeoniae Alba* and *Radix Glycytthizae* are both messenger drugs which can either focus the actions of the formula on a certain area of the body or harmonize and integrate the actions of the other herbs of the formula^6^,^7^. Accumulating evidence has demonstrated that several compositive compounds of herbs in WTD, such as total alkaloids of *Radix Aconiti*, total glycosides and polysaccharides of *Raidix Paeoniae Alba*, and polysaccharides of *Radix Astragali*, may have excellent anti-inflammatory and anti-oxidant activities^8^,^9^. However, monomer pharmacological effects can not present overall efficacy of the whole formula. Although our previous study of network analysis showed that the predicted effectors of WTD might be involved in neuroactive ligand-receptor interaction and calcium signaling pathway^10^, the pharmacological mechanisms of WTD acting on RA have not been fully elucidated.

Because a herbal formula with numerous compositive compounds is too complex to be detected solely by conventional experimental methods, there is an urgent need to develop new and appropriate approaches to address this problem. Network pharmacology, combined with pharmacology and pharmacodynamics, is a novel research field which is implicated in the application of omics and systems biology-based technologies^11^. It clarifies the synergistic effects and the underlying mechanisms of multi-component and multi-target agents by analyzing various networks of the complex and multi-levels interactions^12^,^13^. There are two kinds of approaches in network pharmacology: 1) Bottom-up: Addition of well-known molecular drugs and observation of synergistic effects; 2) Top-down: Reduction of more general formula to its minimal elements that keep its beneficial properties^14^,^15^. As a major tool in network pharmacology, the network analysis based on widely existing databases allows us to form an initial understanding of the action mechanisms within the context of systems-level interactions. Since TCM herbal formula has been considered as a multi-component and multi-target therapeutics which potentially meets the demands of treating a number of complex diseases in an integrated manner, the methodologies of network pharmacology are suitable for pursuing a priori knowledge about the combination rules embedded in formula^16^. Thus, the aim of the current study was to investigate the pharmacological mechanisms of WTD acting on RA through systems approaches integrating drug target prediction, network analysis and experimental validation as shown in Figure 1.

## Results and Discussion

### Putative targets prediction for WTD

Following the drug target prediction by drugCIPHER-CS^14^, we assembled and ranked the 1746 druggable proteins which are often known targets in the DrugBank as putative targets for 451 compositive compounds containing in WTD after deleting redundancy. Of note, there were 101 (5.78%) putative targets identified as known RA-related targets. The detailed information on the predicted drug targets for WTD is described in Supplementary Table S1.

In addition, the top 100 targets were selected as target profiles for each herb since the top 100 targets reach the high prediction accuracy (77.3%) in general^14^. Twenty-three putative targets were common for all five herbs of WTD. More interestingly, *Raidix Paeoniae Alba* and *Radix Glycytthizae* shared more common putative targets with *Radix Aconiti* (both 36/100, 36.00%, Table 1), *Radix Astragali* (62/100, 62.00%, Table 1), and *Herba Ephedrae* (57/100, 57.00%, Table 1), and the two herbs shared the most common potential targets with each other (84/100, 84.00%, Table 1), suggesting their roles in facilitating the effects of other herbs in WTD.

Generally, drug indication for use is determined by functions of its affected targets. In the current study, we collected the known anti-RA drugs with the same targets of herbs in WTD from Therapeutic Target Database^17^ (TTD, http://bidd.nus.edu.sg/group/cjttd/, Aug 25^th^, 2011). As shown in Table 2, five herbs of WTD shared 22 putative targets with known anti-RA drugs, suggesting the possible role of this formula in the treatment of RA. As an autoimmune disease, RA is caused by chronic imbalances between the nervous, endocrine and immune (NEI) systems which constitute systemic properties of an organism^18^. Vagus nerve activity is significantly suppressed in RA patients^19^. Acetylcholine, as the principal vagus neurotransmitter, inhibits inflammation by suppressing the production of pro-inflammatory cytokines that explains why acetylcholine is anti-inflammatory in nature^20^. Growing evidence has demonstrated that aconitine which is the main compositive compounds of *Radix Aconiti* has acetylcholine activity^21^. Consistently, CHRM1, CHRM3 and CHRNA2, which were all putative targets of *Radix Aconiti* shown in Table 2, represent muscarinic acetylcholine receptors and neuronal acetylcholine receptor, and also successful therapeutic targets for RA treatment. In addition, glucocorticoids, an end product of the hypothalamic-pituitaryadrenal axis, are a mainstay treatment for many autoimmune diseases, including RA, because of their potent anti-inflammatory action^22^. Among putative targets of WTD shown in Table 2, NR3C1, the common putative targets for three herbs *Radix Astragali, Herba Ephedrae and Radix Aconiti*, is a glucocorticoid receptor, indicating the glucocorticoid activity of WTD, which was in line with the findings of previous studies^23^. Moreover, pain management is an important component of RA patient care, and opioid analgesics have been extensively used for severe arthritis pain^24^. Accumulating studies have reported the analgesic effects of *Radix Aconiti*^25^, in line with which, we identified three opioid receptors OPRM1, OPRK1 and OPRD1 as putative targets of this herb. From the point of view of immunopathology, RA represents a model for systemic T-cell mediated systemic autoimmunity leading to local cellular and autoantibody mediated chronic inflammation^26^. Thus, targeting of these elements with specific antagonists may interfere with the disease process, reestablishing tolerance and preventing further synovial inflammation. Among the putative targets of WTD shown in Table 2, CD4, IL1B, TNF, NFKB2 and JUN are all involved in immunopathological changes during RA progression. Especially, recent studies have reported that WTD could attenuate the severity of RA or adjuvant arthritis rats by regulating CD4/CD8 ratio and expression levels of several cytokines such as IL1B and TNF^27^,^28^. Taken together, WTD might exert the therapeutic efficacy in the treatment of RA through regulating the expression or activities of its putative targets which are implicated in restoring the balance of NEI system.

### Network analysis

**Figure 2:**
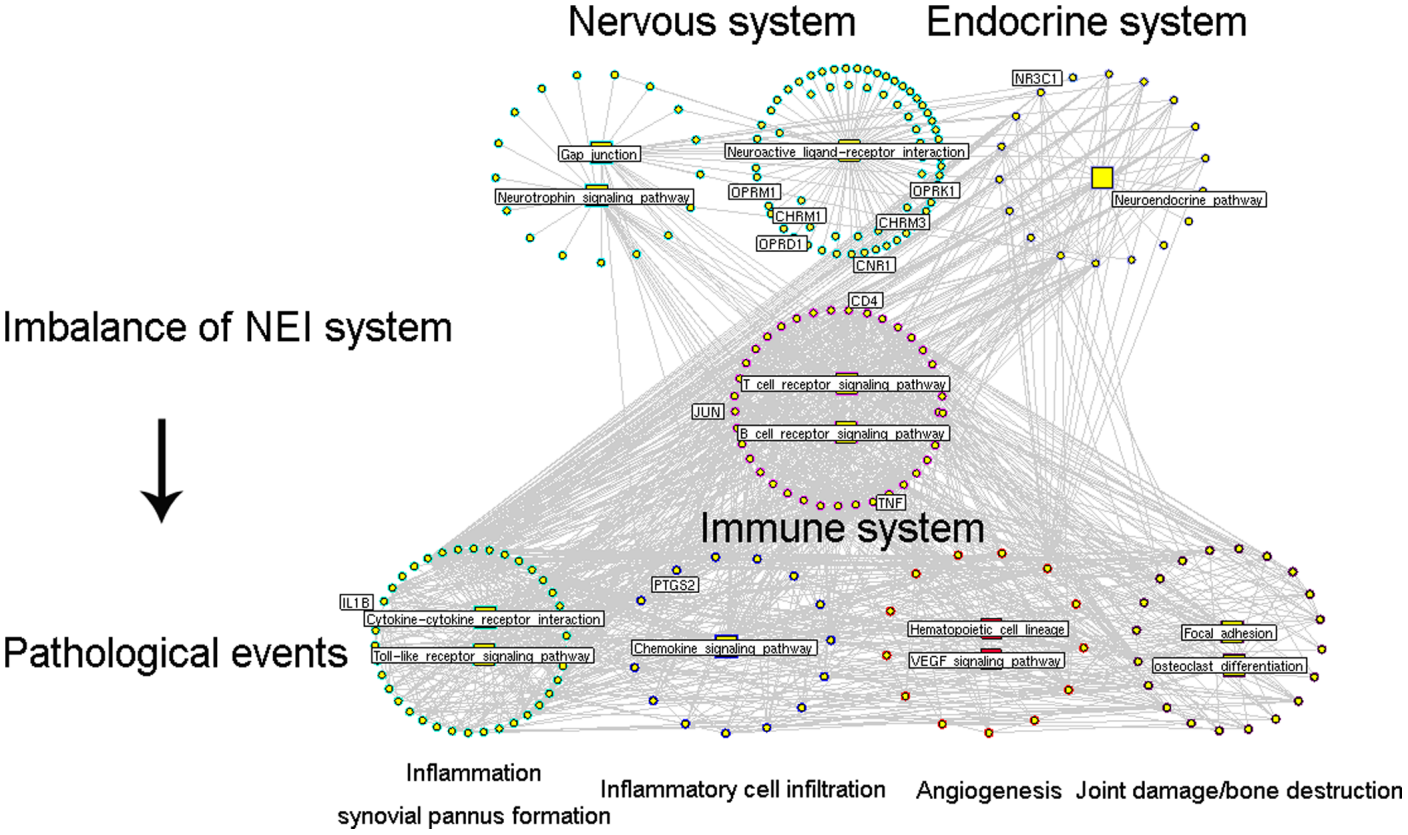
Interaction network of hubs selected from network of putative targets of Wu-tou decoction (WTD) and known RA-related targets. According to their associated biological processes or pathways, these hub nodes were implicated into the imbalance of nervous, endocrine and immune (NEI) system, leading to the main pathological changes during the RA progression.

In order to comprehensively understand the pharmacological mechanisms of WTD, we constructed the interaction network of putative targets and known RA-related targets. According to the previous study of Li et al.^29^, we identified a node as a hub if its degree is more than 2 fold of the median degree of all nodes in a network. Then, we constructed the network of direct interactions among these hub nodes (Please see the interaction network data in Supplementary Table S2). According to their associated biological processes or pathways, these hub nodes were implicated into the imbalance of NEI system, leading to the main pathological changes during the RA progression (Figure 2).

To screen the major hubs, 4 topological features, 'Degree,' 'Node betweenness', 'Closeness' and 'K value' (defined in 'Materials and methods' section), were calculated for each hub in the network. The median values of 'Degree', ' Node betweenness ', 'Closeness' and 'K value' were 21.00, 0.13, 39.34 and 14.00, respectively. Therefore, we determined that hubs with 'Degree'>21.00, ' Node betweenness'>0.13, 'Closeness'>39.34, and 'K value'>14.00 were major hubs. As a result, 121 major hubs were identified (Please see detail information on topological features of 121 major hubs in Supplementary Table S3). After selecting the intersection with putative targets of WTD (Supplementary Table S1), 79 major hubs were identified as candidate targets for this formula.

### Pathway enrichment analysis

**Figure 3:**
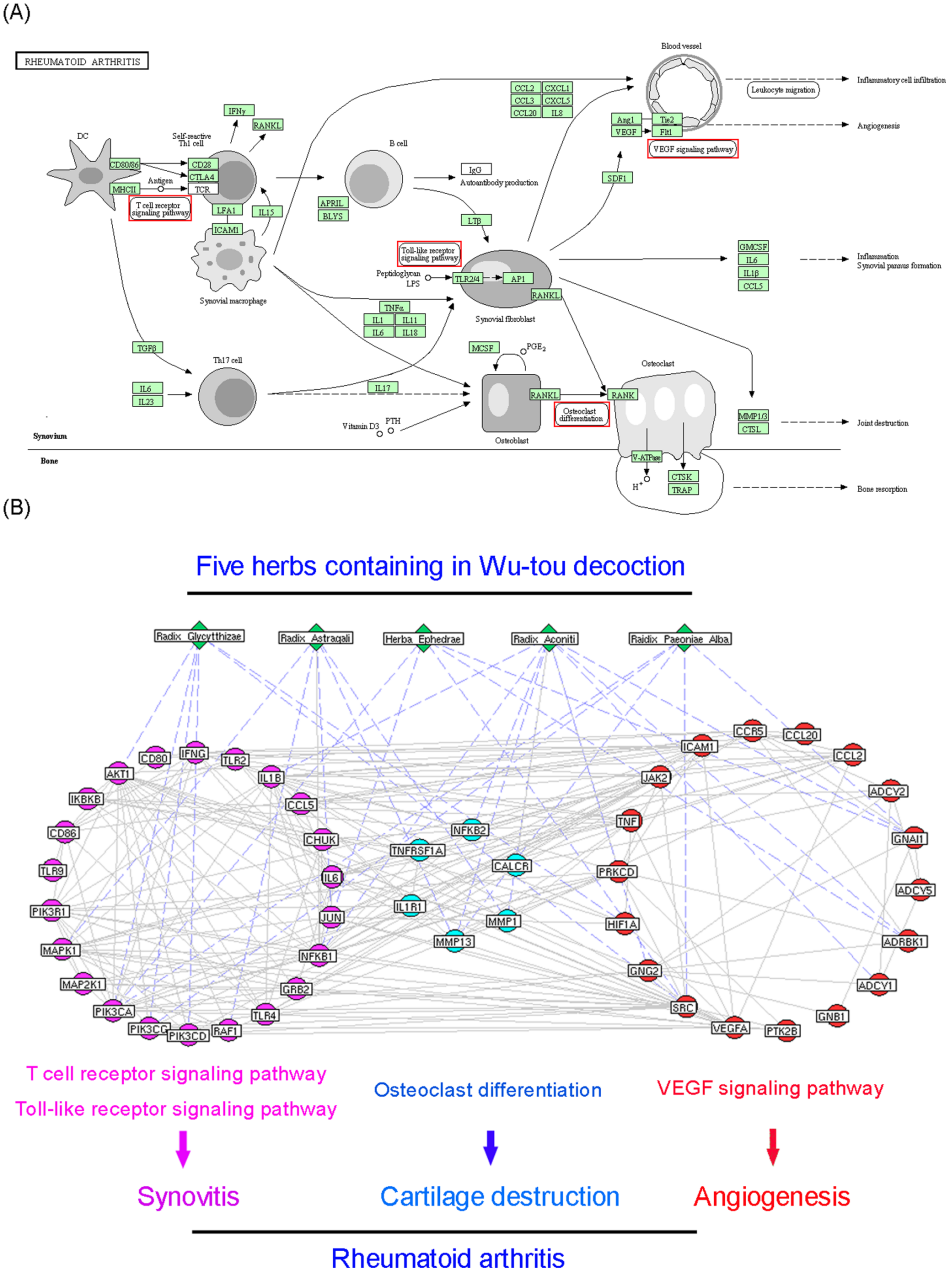
(A) Rheumatoid arthritis pathway (KEGG ID: hsa05323) downloaded from KEGG database. (B) Interaction network of Wu-tou decoction (WTD) candidate targets which are involved into Rheumatoid arthritis pathway and related pathways during the progression of RA.

In the previous section, we found that the putative targets of WTD might play crucial roles in maintaining the balance of NEI system. Here, according to the pathway enrichment analysis (Supplementary Table S4), 79 candidate WTD targets with topological importance in drug-target network were significantly associated with NEI system including Neuroactive ligand-receptor interaction pathway, Progesterone-mediated oocyte maturation pathway, and immune-related pathways (Table 3). Interestingly, targets of WTD were also enriched in the pathway of “Rheumatoid arthritis” (Figure 3A, KEGG ID: hsa05323, http://www.genome.jp/dbget-bin/www_bget?pathway+hsa05323), in which joint damage/bone destruction, inflammation/synovial pannus formation and angiogenesis are three main pathological phenotypes of RA patients. Thus, we speculated that the anti-RA effects of WTD might be associated with the roles of its targets in reversing the imbalance of NEI system and subsequently in the regulation of downstream RA-related pathways, including osteoclast differentiation, T cell receptor signaling pathway, toll-like receptor signaling pathway and VEGF signaling pathway (Figure 3B), which are all associated with patients' phenotypes.

Among candidate WTD targets enriched in the “Rheumatoid arthritis” pathway, three acetylcholine receptors CHRM1, CHRM3 and CHRNA2, glucocorticoid receptor NR3C1, matrix metalloproteinase- (MMP)-1/MMP-13, IL-1β/TNF-α and hypoxia-inducible factor (HIF)-1α/VEGF axes have been indicated as key players of NEI system, osteoclast differentiation, inflammation and VEGF signaling pathway involved during RA progression, respectively. Since the regulatory effects of WTD on these candidate targets have not been fully elucidated, we further performed experimental validation to address this problem based on *in vitro* and *in vivo* systems.

### Experimental validation

#### WTD decreases severity of arthritis in CIA rats

**Figure 4:**
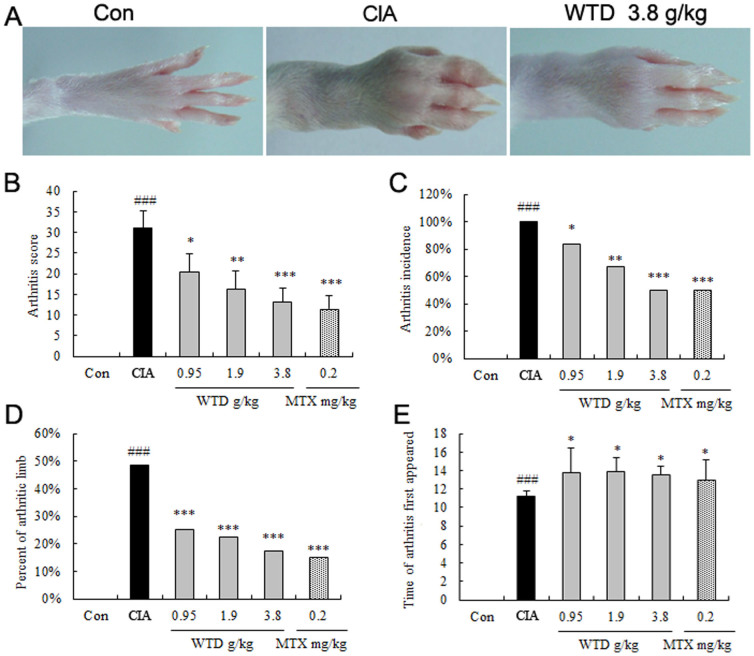
Effects of Wu-tou decoction (WTD) on severity of arthritis in collagen-induced arthritis (CIA) rats. (A) macroscopic evidence of arthritis such as erythema or swelling was markedly observed in vehicle-treated CIA rats, while dose of 3.8 g/(kg**·**day) WTD significantly attenuated arthritis severity in CIA mice; (B) Doses of 0.95 ~ 3.8 g/(kg**·**day) WTD significantly decreased the mean arthritis score in a dose-dependent manner compared with vehicle-treated CIA mice; (C) Doses of 0.95 ~ 3.8 g/(kg**·**day) WTD significantly decreased the arthritis incidence in a dose-dependent manner compared with vehicle-treated CIA rats; (D) Doses of 0.95 ~ 3.8 g/(kg**·**day) WTD significantly decreased the percentage of arthritis limbs in a dose-dependent manner compared with vehicle-treated CIA rats; (E) Doses of 0.95 ~ 3.8 g/(kg**·**day) WTD significantly increased the time of arthritis first appeared compared with vehicle-treated CIA rats. Data are represented as the mean ± S.D. (n = 12). ‘#', P <0.05, comparison with the normal control.‘*’, ‘**', and ‘***', P <0.05, P <0.01, and P <0.001, respectively, comparison with the vehicle control.

To investigate the effect of WTD on arthritis, the CIA model in SD rats was used. Although the disease manifested itself on different days after immunization, we did not observe a relation between clinical response and time of onset of disease. Oral administration of WTD, once a day started when the first clinical signs of disease were beginning, and continued for 21 days. As shown in Figure 4A, macroscopic evidence of arthritis such as erythema or swelling was markedly observed in vehicle-treated CIA rats, while doses of 1.9 g/(kg**·**day) and 3.8 g/(kg**·**day) WTD significantly attenuated arthritis severity in CIA rats. Additionally, the mean arthritis score (all *P* <0.05, Figure 4B), the arthritis incidence (all *P* <0.05, Figure 4C), the percentage of arthritic limbs (all *P* <0.05, Figure 4D) and the time of arthritis first appeared (all *P* <0.05, Figure 4E) in WTD-treated rats were significantly lower than those in vehicle-treated CIA rats with a dose-dependent manner.

#### Radiological and histopathological evaluation

**Figure 5:**
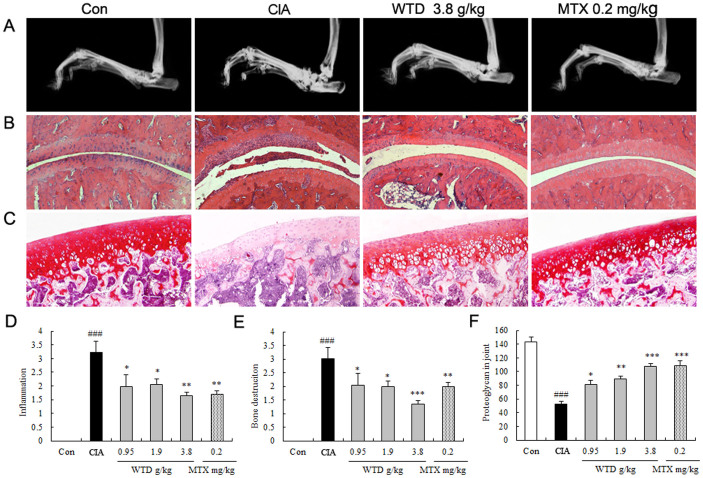
Effect of Wu-tou decoction (WTD) on radiological changes and histologic lesions of CIA rats. (A) shows the clinical manifestation of CIA rats on day 21 after immunization, red swelling in the paws was obviously improved in the WTD-treated group. (B) displays histological observations of the joints in rats (HE staining). (C) shows the results of safranin-O staining in cartilage of joints. (D), (E) and (F) show the inflammation scores, bone destruction score and the content of proteoglycan in joints respectively, as described in methods. Data are represented as the mean ± SD (n = 12). ‘*’, ‘**’, and ‘***', P <0.05, P <0.01, and P <0.001, respectively, comparison with the vehicle control.

Radiological and histopathological evaluation of knee joint sections of vehicle-treated CIA rats revealed inflammatory cell infiltration, synovial hyperplasia and partial bone destruction. In contrast, oral administration of WTD could distinctly reduce the extent of inflammatory cell infiltration, panus formation and bone destruction (Figure 5A and 5B). To elucidate the effects of WTD treatment on inflammation and bone destruction at the radiological and histologic level, inflamed joints were scored with semiquantitative grading scales. As shown in Figure 5D and 5E, the inflammation scores and bone destruction scores in WTD-treated CIA rats were significantly decreased with a dose-dependent tendency in comparison with vehicle-treated CIA rats (all *P* <0.05). MTX also reduced significantly the inflammation score and bone destruction score of inflamed joints compared with vehicle-treated CIA rats (*P <*0.05, Figure 5D and 5E), although this value remained higher than those for WTD-treated groups. Moreover, the content of proteoglycan stained by safranin-O in inflamed joints were increased by WTD dose-dependently (all *P* <0.05, Figure 5C and 5F).

#### WTD reverses the imbalance of NEI systems during RA progression partially by targeting three acetylcholine receptors CHRM1, CHRM3 and CHRNA2, and glucocorticoid receptor NR3C1

**Figure 6:**
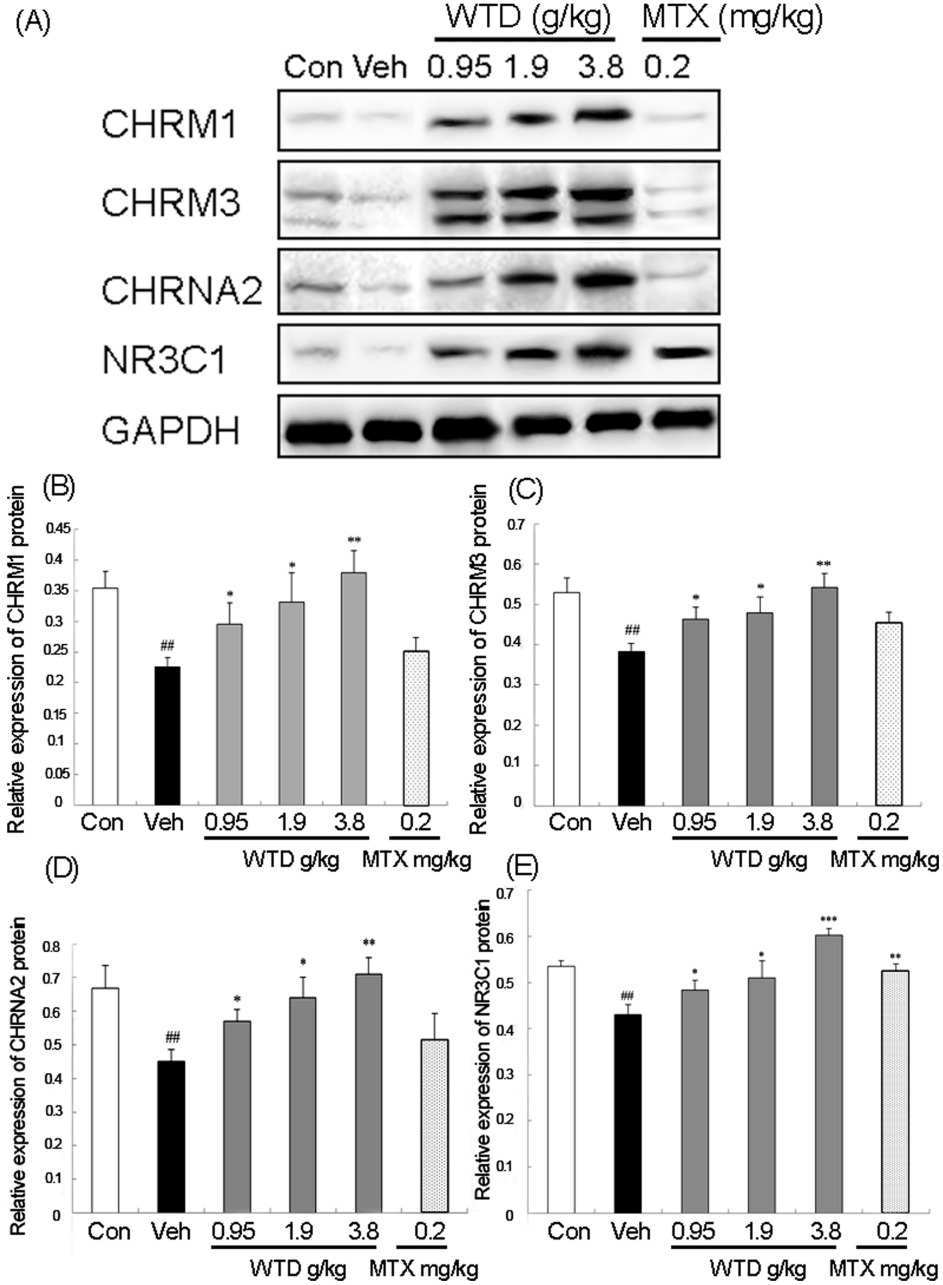
Effect of Wu-tou decoction (WTD) on the expression of three acetylcholine receptors CHRM1, CHRM3 and CHRNA2, and one glucocorticoid receptor NR3C1 in the joint of CIA rats detected by Western blot analysis. Treatment of the rats was the same as the description in Figure 3. (A) Representative blots of CHRM1, CHRM3, CHRNA2 and NR3C1 proteins; (B)~(E) Relative expression levels of CHRM1, CHRM3, CHRNA2 and NR3C1 proteins in different groups. Data are represented as the mean ± S.D. ‘^###^', P <0.001, comparison with the normal control. ‘*’, ‘**’, and ‘***', P <0.05, P <0.01, and P <0.001, respectively, comparison with the vehicle control.

The imbalances of NEI systems have been regarded as one of the main causes of occurrence and progression of RA. In the current study, Western blot analysis was performed and the results in Figure 6 showed that the protein expression levels of three acetylcholine receptors CHRM1, CHRM3 and CHRNA2, and glucocorticoid receptor NR3C1 in inflamed joints of CIA rats were distinctly decreased compared with normal controls (all P <0.01, Figure 6), which were all reversed by the treatment of WTD with a dose-depend manner (all P <0.05, Figure 6). In addition, we found that Methotrexate, which is considered as the 'anchor drug' in RA treatment, could not change the expression levels of three acetylcholine receptors, implying there might be no drug-target interactions between Methotrexate and acetylcholine receptors. Moreover, Methotrexate significantly increased the expression of NR3C1 protein in the inflamed joints of CIA rats compared with vehicle controls (all P <0.01, Figure 6E), but had no differences with statistical significance when compared with WTD-treated groups. These findings suggest that WTD may reverse the imbalance of NEI systems during RA progression partially by regulating its candidate targets including CHRM1, CHRM3, CHRNA2 and NR3C1.

#### WTD reverses the main pathological events of RA by targeting MMP-1/MMP-13, IL-1β/TNF-α and HIF-1α/VEGF signal axes in vitro and in vivo systems

**Figure 7:**
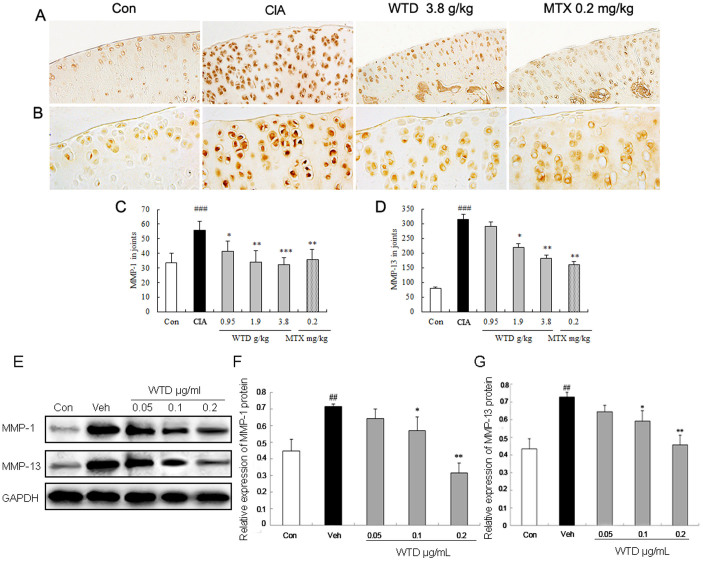
Effect of Wu-tou decoction (WTD) on the expression of MMP-1 and MMP-13 in the joint of CIA rats and in human fibroblast-likesynoviocytes of rheumatoid arthritis (HFLS-RA). Treatment of the rats was the same as the description in Figure 3. (A) and (B) respectively showed the few positive signals for MMP-1 and MMP-13 in normal control rats, while MMP-1 and MMP-13 were strongly expressed in cartilage of the CIA rats. (C) and (D) Doses of 0.95 ~ 3.8 g/(kg**·**day) WTD significantly decreased the expression of MMP-1 and MMP-13 compared with vehicle-treated CIA rats. Data are represented as the mean ± S.D. (n = 12). '^###^', P <0.001, comparison with the normal control. ‘*', ‘**', and ‘***', P <0.05, P <0.01, and P <0.001, respectively, comparison with the vehicle control. (E) Representative blots of MMP-1 and MMP-13 proteins detected by western blot analysis; (F)~(G) Relative expression levels of MMP-1 and MMP-13 proteins in different groups. Data are represented as the mean ± S.D. ‘^##^', P <0.01, comparison with the control cells.‘*' and ‘**', P <0.05 and P <0.01, respectively, comparison with the IL-1β-induced vehicle control.

To obtain insights into the mechanisms of the inhibitory effects of WTD on cartilage-destruction in inflamed joints of CIA rats, the expression levels of MMP-1 and MMP-13 proteins in inflamed joints in different groups were detected by immunohistochemistry (Figure 7). Compared with vehicle-treated CIA rats, doses of 0.95 ~ 3.8 g/(kg**·**day) WTD significantly reduced the expression of MMP-1 (all P <0.05, Figure 7A and C) and MMP-13 (all P <0.05, Figure 7B and D). Methotrexate also significantly reduced the expression of MMP-1 and MMP-13 proteins in the inflamed joints of CIA rats compared with vehicle controls (all P <0.01, Figure 7A~D), although this value remained higher than those for WTD-treated groups (P <0.05, Figure 7A~D). These findings were all consistent with the data based on in vitro cultured human fibroblast-likesynoviocytes of RA (HFLS-RA) detected by western blot analysis as shown in Figure 7E~G. Under the pathological conditions, especially in the progression of RA, the destruction of extracellular matrix (ECM) components may cause the impairment of joint functions^30^. Among ECM components, MMPs have been demonstrated to play a central role in this process. MMP-1 and MMP-13 expression levels have been found to be increased in synovial tissues and fluid from RA patients and their aberrant expression may be implicated in the degradation of connective tissue components in cartilage with RA^31^,^32^. In the present study, our data showed that the treatment of WTD significantly interfered with the RA-augmented expression of MMP-1 and MMP-13 proteins *in vivo* and *in vitro* systems, suggesting that WTD may prevent cartilage-destruction during the progression of RA by targeting MMP-1/MMP-13 axis.

**Figure 8:**
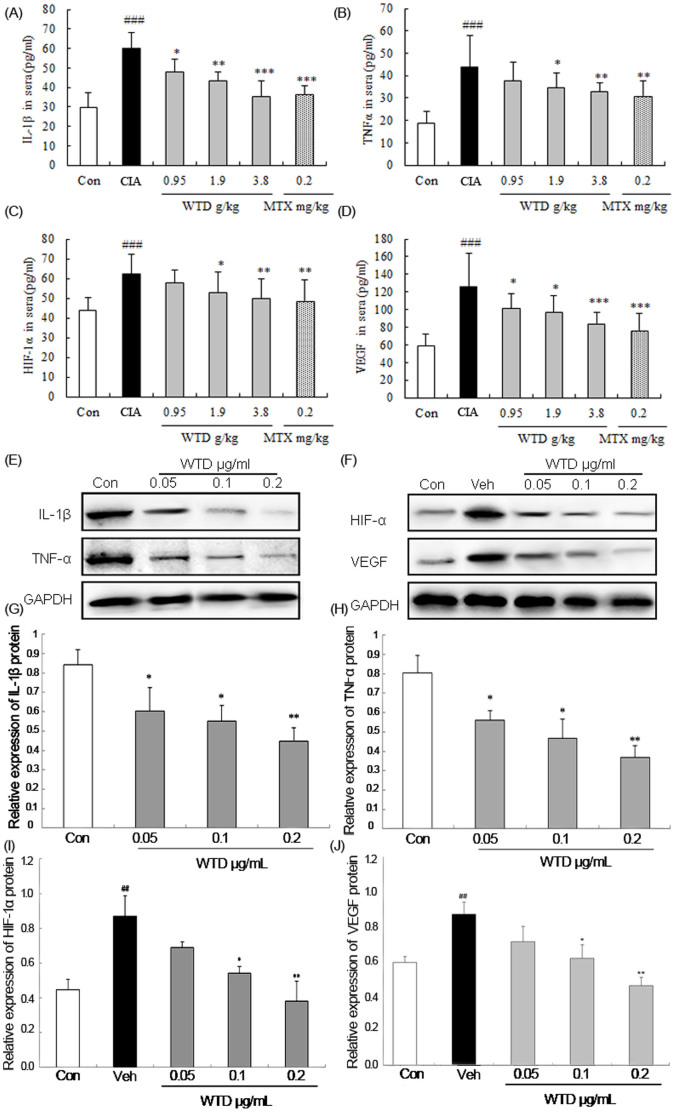
Effect of Wu-tou decoction (WTD) on IL-1β, TNFα, HIF-1α and VEGF in sera of CIA rats and in human fibroblast-likesynoviocytes of rheumatoid arthritis (HFLS-RA). Treatment of the rats was the same as the description in Figure 3. (A-D). Data are represented as the mean ± SD (n = 12). ‘^###^', P <0.001, comparison with the normal control. ‘*', ‘**', and ‘***', P <0.05, P <0.01, and P <0.001, respectively, comparison with the vehicle control. (E)~(F) Representative blots of IL-1β, TNFα, HIF-1α and VEGF proteins detected by western blot analysis; (G)~(J) Relative expression levels of IL-1β, TNFα, HIF-1α and VEGF proteins in different groups. Data are represented as the mean ± S.D. ‘^##^', P <0.01, comparison with the control cells. ‘*’ and ‘**', P <0.05 and P <0.01, respectively, comparison with the IL-1β-induced vehicle control.

In addition, we also detected the expression levels of IL-1β, TNF-α, HIF-α and VEGF proteins in the sera of CIA rats and in HFLS-RA of different treatment groups by ELISA assay and western blot analysis, respectively, in order to investigate the possible mechanisms of WTD treatment on attenuating synovitis and angiogenesis in joints during RA progression. As a result, WTD treatments also markedly reduced the expression levels of IL-1β, TNF-α, HIF-α and VEGF proteins both in the sera of CIA rats and in HFLS-RA with a dose-dependent tendency (all *P* <0.05, Figure 8). Various inflammatory cytokines such as IL-1β, IL-6, and TNF-α have been found to be upregulated in synovial fluid from RA patients^33^. Among them, IL-1β and TNF-α are known to be pivotal factors, since they strictly enhance the production of pro-MMPs and an inflammatory mediator, prostaglandin E2 (PGE2), in synoviocytes and chondrocytes^34^. Here, our data shown that the treatment of WTD could effectively reduce the expression of IL-1β and TNF-α at protein level both in the sera of CIA rats and in HFLS-RA, implying that WTD may suppress the synovitis in RA by targeting IL-1β/TNF-α axis. More interestingly, angiogenesis appears to play an important role in the pathogenesis of RA^35^. Recent studies have confirmed the overexpression of VEGF in sera and synovial fluid from RA patients. HIF-1α, as an essential regulatory factor of the transcription of the VEGF gene, has been demonstrated to be involved in the angiogenesis of RA^36^. In the current study, we found that the upregulation of both HIF-1α and VEGF proteins in sera from CIA rats and in HFLS-RA could be significantly suppressed by the treatment of WTD, indicating that this formula may have an anti-angiogenesis effect for RA by targeting HIF-1α/VEGF.

In conclusion, the current study provided an integrative analysis by combining drug target prediction and network analysis to understand the pharmacological mechanisms of WTD acting on RA. Further experimental validation also offered the convincing evidence that WTD may attenuate RA partially by restoring the balance of NEI system and subsequently reversing the pathological events during RA progression by regulating MMP-1/MMP-13, IL-1β/TNF-α and HIF-1α/VEGF signal axes.

## Methods

### Data preparation

#### Compositive compounds of each herb in WTD

Compositive compounds of each herb in WTD were obtained from TCM Database@Taiwan^37^ (http://tcm.cmu.edu.tw/, Updated in 2012-06-28), which is currently the largest non-commercial TCM database worldwide. TCM Database@Taiwan is based on information collected from Chinese medical texts and scientific publications, and contains more than 20,000 pure compounds isolated from 453 TCM herbs. In total, we collected the structural information of 22 compounds for *Radix Aconiti*, 122 compounds for *Herba Ephedrae*, 39 compounds for *Radix Astragali*, 65 compounds for *Raidix Paeoniae Alba* and 203 compounds for *Radix Glycytthizae*. The detailed information on these compositive compounds of each herb in WTD is described in Supplementary Table S5.

#### Known RA-related targets

Known RA-related targets were obtained from four existing resources: (1) DrugBank database^38^ (http://www.drugbank.ca/, version: 3.0). We only used those drug-target interactions whose drugs are FDA approved for the treatment of RA and whose targets are human genes/proteins. In total, we obtained 58 known RA-related targets. (2) The Online Mendelian Inheritance in Man (OMIM) database^39^ (http://www.omim.org/, Last updated: October 31, 2013). We searched the OMIM database with a keyword “rheumatoid arthritis” and found 7 known RA-related targets: CD244, HLA-DR1B, MHC2TA, NFKBIL1, PAD, SLC22A4, and PTPN8. (3) Genetic Association Database (GAD)^40^ (http://geneticassociationdb.nih.gov/, Last updated: August 18, 2013). We used a keyword “rheumatoid arthritis” to search the GAD database. In total, we obtained 82 known RA-related targets whose association with RA was shown “Y”. (4) Kyoto Encyclopedia of Genes and Genomes (KEGG) Pathway Database^41^ (http://www.genome.jp/kegg/, Last updated: October 16, 2012). In total, we obtained 92 known RA-related targets which appear on the RA pathway (KEGG ID: map05323) in the KEGG database. The detailed information on these known therapeutic targets is described in Supplementary Table S6. After deleting redundancy, there were 208 known RA-related targets collected in this study.

#### Protein-protein interaction (PPI) data

PPI data were imported from eight existing PPI databases including Human Annotated and Predicted Protein Interaction Database (HAPPI)^42^, Reactome^43^, Online Predicted Human Interaction Database (OPHID)^44^, InAct^45^, Human Protein Reference Database (HPRD)^46^, Molecular interaction Database (MINT)^47^, Database of Interacting Proteins (DIP)^48^, and PDZBase^49^. The detailed information on these PPI databases is described in Supplementary Table S7.

### Drug target prediction for WTD

The putative targets of WTD's compositive compounds were predicted by drug-CIPHER-CS presented by Zhao and Li^14^. Based on two hypotheses: (i) drugs with similar chemical structure usually bind functionally related proteins and (ii) functional relationship between the proteins can be measured by their distance in the protein interaction network, drugCIPHER-CS achieves good prediction performance and can infer drug targets in the genome-wide scale. This method calculates the likelihood of the interactions of drug-target based on the correlation between the query drug's structure similarity vector with the drug space and the candidate gene's functional similarity vector with the target space. For a query compound, drug-CIPHER-CS prioritizes the proteins in the PPI network according to the order of the decreasing drug target interaction likelihood, and the candidate proteins with high likelihood will be hypothesized as the putative targets.

### Network construction

We first constructed a interaction network for known RA-related targets and putative drug targets of WTD based on their interaction data obtained from eight existing PPI databases as mentioned above. Then, we applied Navigator software (Version 2.2.1) to visualize the interaction network.

#### Defining network topological feature set

For each node i in interaction network, we defined four measures for assessing its topological property: (1) 'Degree' is defined as the number of links to node i; (2) 'Node betweenness' is defined as the number of shortest paths between pairs of nodes that run through node i. (3) ‘Closeness' is defined as the inverse of the farness which is the sum of node i distances to all other nodes. The Closeness centrality can be regarded as a measure of how long it will take to spread information from node i to all other nodes sequentially. Degree, node betweenness and closeness centralities can measure a node's topological importance in the network. The larger a node's degree/node betweenness/closeness centrality is, the more important the node is in the interaction network^50^. (4) K-core analysis is an iterative process in which the nodes are removed from the networks in order of least-connected^51^. The core of maximum order is defined as the main core or the highest k-core of the network. A k-core sub-network of the original network can be generated by recursively deleting vertices from the network whose degree is less than k. This results in a series of sub-networks that gradually reveal the globally central region of the original network. On this basis, 'K value' is used to measure the centrality of node i.

### Pathway enrichment analysis for candidate WTD targets

We used Database for Annotation, Visualization and Integrated Discovery^52^ (DAVID, http://david.abcc.ncifcrf.gov/home.jsp,version 6.7) for GO enrichment analysis. We also performed pathway enrichment analysis using pathway data obtained from the FTP service of KEGG^53^ (Kyoto Encyclopedia of Genes and Genomes, http://www.genome.jp/kegg/, Last updated: Oct 16, 2012).

### Experimental validation

The study was approved by the Research Ethics Committee of Institute of Chinese Materia Medica, China Academy of Chinese Medical Sciences, Beijing, China. All animals were treated in accordance with the guidelines and regulations for the use and care of animals of the Center for Laboratory Animal Care, China Academy of Chinese Medical Sciences.

#### Animals

A total of 72 male SD rats (160 ~ 180 g) were purchased from Experimental Animal Center, Academy of Military Medical Sciences (production license No: SCXK 2002-001). All rats were maintained in a room equipped with an air-filtering system, and the cages and water were sterilized.

#### Cell culture

HFLS-RA (Cell Applications, USA) was used for the in vitro experimental validation in the current study. The cells were cultured in sterile synoviocyte growth medium (Cell Applications, USA) supplemented with 100 U/mL 1 penicillin, 80 U/mL 1 streptomycin, 2 mM Gln-glutamine, and were maintained at 37°C in a humidified 5% CO_2_ incubator. HFLS–RA were used at passage numbers 4 to 8 in this study.

#### Induction of CIA

CIA was induced as our previously reported^54^,^55^,^56^. Briefly, bovine type II collagen (Chondrex, Redmond, WA, USA) was dissolved in 0.1 M acetic acid overnight at 4°C. This was emulsified in an equal volume of incomplete Freund's adjuvant (IFA, Chondrex, Redmond, WA, USA). The rats were immunized intradermally at the base of the tail with 100 μl of emulsion containing 100 μg of type II collagen. On day 7, rats were boosted intraperitoneally with 100 μg type II collagen in IFA.

#### Treatment and groups

According to the original composition of WTD recorded in Chinese Pharmacopoeia 2010 edition, WTD was prepared using the following procedure. The crude drugs of *Radix Aconiti* 6 g, *Herba Ephedrae* 9 g, *Radix Astragali* 9 g, *Raidix Paeoniae Alba* 9 g and *Radix Glycytthizae* 9 g were immersed in 2 litres of water for 2 h and then decocted to boil for 1 h. The decoction was filtered through four layers of gauze. Next, the drugs were boiled once again for 0.5 h with 2litres of water and the decoction was filtrated out with the above method. Finally, the extraction solution was made to a concentration of 1 g crude drug/mL. To clarify the chemical composition of WTD, UPLC–Q-TOF-MS analysis was conducted to identify its major compounds. The detailed strategy and results of the identification were provided in our previously published paper^57^.

For in vivo experimental validation, the route of WTD delivery was oral administration. Treatment was given daily for a period of 21 days. The dosage selection for WTD [3.8 μg/(kg·day)] was nearly equivalent to RA patient dosage daily (42 g/person/day). SD rats were divided into 6 groups with the equal number (n = 12): normal control group (Normal), CIA model control group (Vehicle), CIA rats treated with 0.95 g/(kg·day) WTD (WTD-low), 1.9 g/(kg·day) WTD (WTD-middle), 3.8 g/(kg·day) WTD (WTD-high), and 0.2 mg/kg methotrexate (MTX).

For in vitro experimental validation, HFLS-RA were then incubated with different concentrations of WTD (0.05, 1.0 and 2.0 μg/mL) for 24 h.

#### Severity assessment of arthritis

Rats were observed once every day after primary immunization. Arthritis severity was evaluated by arthritis score, arthritis incidence, percentage of arthritic limbs and the time of arthritis first appeared which were performed by two independent, blinded observers. The arthritis score was the total of the scores for all 4 limbs (maximum possible arthritis score 80). Arthritis incidence values are the number positive/total number in group. In addition, the number of arthritic limbs of individual rats were counted and added to represent the number of arthritic limbs in a group. The percentage of arthritic limbs in a group was calculated as following formula:
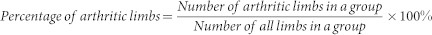


Moreover, the time of arthritis first appeared refered to the first day of the onset of the clinical symptoms of arthritis observed.

#### Histology and histologic scoring

Rats were sacrificed by cervical dislocation on day 21 after first immunication. Both hind limbs including the paws, ankles, and knees, were dissected, fixed immediately for 24 h in 4% paraformaldehyde, decalcified in 10% EDTA for up to 2 month at 4°C, and embedded in paraffin. Tissue sections (4 μm) were mounted on common slides for staining with hematoxylin and eosin (H&E) or safranin-O. All sections were randomized and evaluated by two trained observers who were blinded to the treatment groups and the arthritis severity of each rat. Minor differences between observers were resolved by mutual agreement. The data was expressed as mean inflammation score. All scores were based on a scale of 0–3, as previously described^58^.

#### Radiological observation

At the end of the experiment, rats were sacrificed and the left hind paws were radiographed with a digital mammography system (Planmed, Finland). Radiographs of ankle and tarsus joints of each rat were evaluated for bone destruction on a scale of 0 = normal, 1 = mild changes, 2 = moderate changes, and 3 = severe changes, respectively^59^. Two observers blind to treatment assignment and with significant experience in reading and rating radiographs for patients with RA evaluated the radiographs. A total radiological score was obtained by summing the scores awarded to the left hind paw by both observers, giving a maximum score of 6 per rat for each radiological parameter.

#### Immunohistochemical staining

Paraffin sections (5 μm) of tissue from the knee and ankle joints were mounted on poly-L-lysine-coated slides. Immunolocalizations of MMP-1 and MMP-13 in the joints were carried out with commercial Polink-2 plus Polymer HRP Detection System For Goat Primary Antibody kits (Golden Bridge International Inc., Mukilteo, WA, USA) according to the manufacturer's instructions. The paraffin sections were dewaxed by routine method and incubated for 10 min with 3% H_2_O_2_. Each section was incubated with normal goat serum for 20 min at room temperature, and then with primary antibodies against rat MMP-1 (Abcam, Cambridge, UK) and MMP-13 (Abcam, Cambridge, UK) respectively overnight at 4°C. After incubation with Polymer Helper for 20 min at 37°C, sections were reacted with poly-HRP anti-goat IgG for 20 min at 37°C. The sections were then stained with 3, 3-diaminobenzidine (Sigma, St. Louis, MO, USA) and counterstained with hematoxylin. For the control staining, PBS was used instead of the primary antibodies.

Specimens were examined using a Leica image analyzer and analyzed by computer image analysis (Leica Microsystem Wetzlar Gmbh., Wetzlar, Germany) in a blinded manner. To localize and identify areas with positively stained cells, ten digital images per specimen of synovium from a knee or ankle joint were recorded, and quantitative analysis was performed according to the color cell separation. The results are expressed as the mean region of interest, representing the percentage of area covered with positively stained cells per image at a magnification of ×400.

#### Enzyme-linked immunosorbant assay

Sera from the rats on day 22 of arthritis were obtained and stored at −80°C until use. The amounts of IL-1β, TNF-α, HIF-1α and VEGF in sera were detected by ELISA assay (R&D system, Minneapolis, MN, USA) according to the manufacturer's protocol and absorbance was measured at 450 nm. All experiments were done in triplicate.

#### Western blot analysis

The Western blot protocol and semiquantitative analysis were carried out following the protocol of our previous studies^54^,^56^. The following antibodieswere used: IL-1β antibody (rabbit monoclonal antibody, dilution 1:20000, Abcam.UK), TNF-α antibody (rabbit polyclonal antibody, dilution 1:100, Abcam. UK), VEGF antibody (rabbit monoclonal antibody, dilution 1:1000, Abcam. UK), HIF-1α antibody (rabbit monoclonal antibody, dilution 1:1000, Abcam. UK), MMP-1 antibody (rabbit monoclonal antibody, dilution 1:1000, Abcam. UK), MMP-13 antibody (rabbit monoclonal antibody, dilution 1:500, Abcam. UK), CHRM1 antibody (rabbit polyclonal antibody, dilution 1:500, Abcam. UK), CHRM3 antibody (rabbit monoclonal antibody, dilution 1:1000, Abcam. UK), CHRNA2 antibody (rabbit monoclonal antibody, dilution 1:1000, Abcam. UK), NR3C1 antibody (rabbit monoclonal antibody, dilution 1:50000, Abcam. UK). All experiments were done in triplicate. Mean normalized protein expression ± SEM was calculated from independent experiments.

#### Statistical analysis

The software of SPSS version13.0 for Windows (SPSS Inc, Chicago, IL, USA) and SAS 9.1 (SAS Institute, Cary, NC) was used for statistical analysis. Continuous variables were expressed as 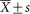. Arthritis incidence and percentage of arthritic limbs were analyzed by a chi-square test. Arthritis index and pathological score were analyzed with non-parametric statistics (Kruskal-Wallis test). Other data were analyzed by one-way ANOVA followed by LSD test. Differences were considered statistically significant when *P* was less than 0.05.

## Author Contributions

S.L. and N.L. engaged in study design and coordination, material support for obtained funding, and supervised study. Y.Z. performed network analysis, designed the experimental validation and drafted the manuscript. M.B. and B.Z. performed drug target prediction. C.L., Y.S., D.W., Y.J. and Q.G. carried out the experiment validation. All authors reviewed the manuscript.

## Supplementary Material

Supplementary Information Table S1

Supplementary Information Table S2

Supplementary Information Table S3

Supplementary Information Table S4

Supplementary Information Table S5

Supplementary Information Table S6

Supplementary Information Table S7

## Figures and Tables

**Figure 1:**
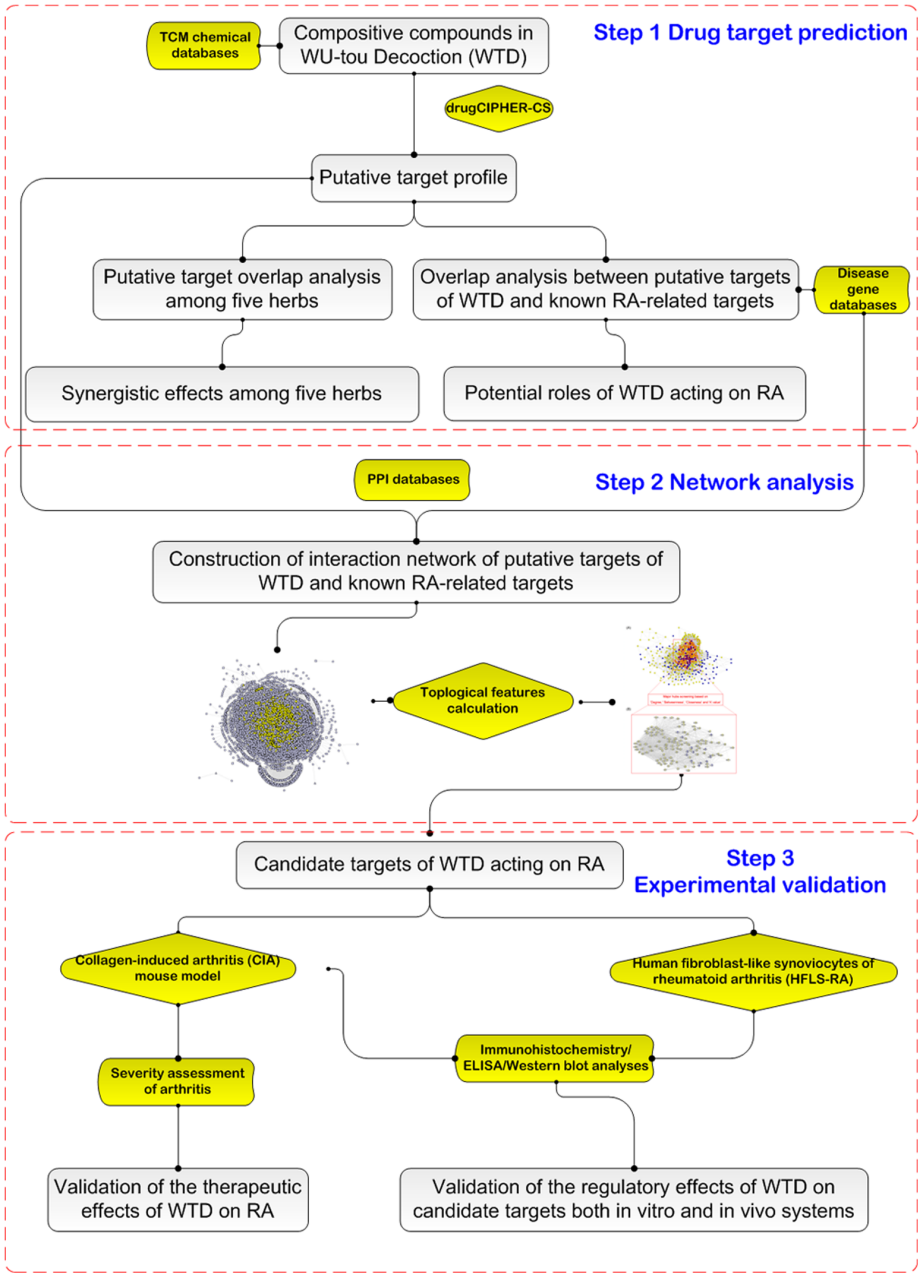
A schematic diagram of the systematic strategies for uncovering the pharmacological mechanisms of herbal formula Wu-tou decoction acting on RA.

**Table 1:** Putative target overlaps among five herbs of Wu-tou decoction

Herbs	Radix Aconiti (100)	Radix Astragali (100)	Herba Ephedrae (100)	Raidix Paeoniae Alba (100)	Radix Glycytthizae (100)
*Radix Aconiti* (100)	-	36	48	36	36
*Radix Astragali* (100)	36	-	48	62	62
*Herba Ephedrae* (100)	48	48	-	57	57
*Raidix Paeoniae Alba* (100)	36	62	57	-	84
*Radix Glycytthizae* (100)	36	62	57	84	-

**Table 2:** Potential target overlaps among five herbs of Wu-tou decoction (WTD)

Herbs in WTD	Target name	Target symbol	Known drug	Indication	Target classification
*Radix Aconiti*	Nuclear factor NF-kappa-B	NFKB2	Curaxin CBLC102; GMX1777; PG-490-88; Sulfasalizine; Tyloxapol;	rheumatoid arthritis	Clinical trial target
*Radix Astragali*	Transcription factor AP-1	JUN	T-5224	rheumatoid arthritis	Clinical trial target
*Radix Aconiti*	Metabotropic glutamate receptor 1	GRM1	AZD-9272; AZD8529; PF-1913539;	pain	Clinical trial target
*Radix Astragali/Radix Aconiti*	T-cell surface glycoprotein CD4	CD4	Blinatumomab;Anti-CD4	rheumatoid arthritis	Clinical trial target
*Radix Aconiti*	Beta-2 nAChR	CHRNB2	ABT-894	pain	Discontinued target
*Radix Aconiti*	Muscarinic acetylcholine receptor M1	CHRM1	Benztropine; Biperiden; Clidinium; Cycrimine; Darotropium; Darotropium + 642444; Dicyclomine; Ethopropazine; GSK1034702; GSK573719; GSK961081; Glycopyrrolate; Oxyphenonium; Pirenzepine; Propantheline; Revatropate; Sabcomeline hydrochloride; Talsaclidine fumarate; Talsaclidine isomer; Trihexyphenidyl; Xanomeline tartrate;	pain	Successful target
*Radix Aconiti*	Muscarinic acetylcholine receptor M3	CHRM3	Cevimeline; Darifenacin; Diphemanil Methylsulfate; LAS-34273; Oxybutynin; Revatropate; Solifenacin; Succinylcholine; Tiotropium; Tolterodine;	pain	Successful target
*Raidix Paeoniae Alba/Herba Ephedrae*	Prostaglandin G/H synthase 1	PTGS1	Suprofen; Salsalate	rheumatoid arthritis and osteoarthritis	Successful target
*Raidix Paeoniae Alba/Herba Ephedrae*	COX-2	PTGS2	Tolmetin;Tiaprofenic acid	pain and rheumatoid arthritis	Successful target
*Radix Glycytthizae/Radix Astragali/Radix Aconiti*	Aldose reductase	AKR1B1	Zenarestat;Sulindac	rheumatoid arthritis	Successful target
*Radix Aconiti*	Mu-type opioid receptor	OPRM1	Alfentanil; Alvimopan; Anileridine; Buprenex; Buprenorphine; Diphenoxylate; Fentanyl; GNTI; GSK1521498; LY-25582; Levomethadyl Acetate; Methadyl Acetate; Methylnaltrexone; Naloxone; Oxymorphone; Remifentanil; TD-1211; Tramadol ER;	pain	Successful target
*Radix Aconiti*	Kappa-type opioid receptor	OPRK1	Asimadoline; Dezocine; GNTI;	pain	Successful target
*Radix Aconiti*	Delta-type opioid receptor	OPRD1	BIO-306; Butorphanol; Codeine; Divers drug; Hydrocodone; Hydromorphone; Loperamide; Nalbuphine; Naltrexone; Oxycodone;	pain	Successful target
*Radix Aconiti*	Voltage-gated sodium channel subunit alpha Nav1.8	SCN10A	SPI-860; Tetracaine	pain	Successful target
*Radix Aconiti*	Tachykinin 1 receptor	TACR1	AZD2624; Aprepitant; CS-003; Casopitant; DA-5018; DNK-333; Ezlopitant; GSK 679769; GSK1144814; GSK424887; L-759274; Orvepitant; R 673; SLV-317; SLV-323; TAK-637; Vestipitant; Zunrisa/Rezonic;	pain	Successful target
*Raidix Paeoniae Alba/Radix Glycytthizae/Radix Astragali/Herba Ephedrae/Radix Aconiti*	Bile acid receptor	NR1H4	Guggulsterone	osteoarthritis	Successful target
*Radix Astragali/Herba Ephedrae/Radix Aconiti*	Glucocorticoid receptor	NR3C1	Amcinonide; Betamethasone; Budesonide; Dexamethasone; Flunisolide; Fluorometholone; Fluticasone; Fluticasone Propionate; GW685698X; GW870086X; ISIS-GCCRrx; Loteprednol Etabonate; Medrysone; Methylprednisolone; Mometasone; ORG 34517/34850; Prednisone;	rheumatoid arthritis	Successful target
*Radix Aconiti*	Cannabinoid receptor 1	CNR1	AVE1625; AZD1175; AZD1704; AZD1940; AZD2207; CB1 antagonist; CBD cannabis derivative; CP-945598; Dianicline+rimonabant; JD-5037; KDS-2000; Marinol; Rimonabant; Rimonbant; SLV-319; TM38837; Taranabant; Tebipenem; ZY01;	pain	Successful target
*Radix Aconiti*	Neuronal acetylcholine receptor subunit alpha-2	CHRNA2	Carbachol; Decamethonium; Doxacurium chloride; Levallorphan; Metocurine; Metocurine Iodide; Mivacurium; Pipecuronium; Rocuronium; Tubocurarine; Vecuronium;	pain	Successful target
*Radix Aconiti*	Interleukin-1 beta	IL1B	Canakinumab; Celastrol; Gallium nitrate; Glucosamine; Ibudilast;	osteoarthritis	Successful target
*Raidix Paeoniae Alba/Radix Glycytthizae/Herba Ephedrae*	Tumor necrosis factor	TNF	Etanercept/Adalimumab/Infliximab/	rheumatoid arthritis	Successful target
*Herba Ephedrae*	Voltage-dependent N-type calcium channel subunit alpha-1B	CACNA1B	Cilnidipine; Ralfinamide; Ziconotide;	pain	Successful target

**Table 3:** Top 10 pathways associated with 79 candidate targets of Wu-tou decoction (WTD) according to the enrichment analysis based on KEGG pathway

KEGG_ID	Term	Gene_symbol	P (Bonferroni correction)
hsa04062	Chemokine signaling pathway	AKT1/CCL5/RAF1/CCL20/PIK3R1/ADRBK1/NFKB1/ADCY1/JAK2/PIK3CA/PRKCD/PIK3CG/CCL2/GNAI1/ADCY2/GNG2/GRB2/PTK2B/IKBKB/MAPK1/PIK3CD/CCR5/CHUK/ADCY5/MAP2K1/GNB1	3.16E-21
hsa04620	Toll-like receptor signaling pathway	AKT1/IL6/CCL5/CD86/PIK3R1/NFKB1/TLR9/PIK3CA/PIK3CG/JUN/CD80/TLR4/IKBKB/MAPK1/TLR2/PIK3CD/IL1B/CHUK/MAP2K1	5.20E-18
hsa04380	Osteoclast differentiation	AKT1/PIK3R1/NFKB2/IL1R1/NFKB1/PIK3CA/PIK3CG/JUN/GRB2/IKBKB/IFNG/MAPK1/MMP1/MMP13/CALCR/PIK3CD/FCGR3A/IL1B/CHUK/TNFRSF1A/MAP2K1	2.74E-16
hsa04660	T cell receptor signaling pathway	AKT1/RAF1/PIK3R1/NFKB1/CTLA4/PIK3CA/PIK3CG/JUN/CD4/GRB2/IKBKB/IFNG/MAPK1/PRKCQ/PIK3CD/CHUK/MAP2K1	7.40E-15
hsa05323	Rheumatoid arthritis	CD80/VEGFA/IL6/CCL5/CD86/TLR4/CCL20/MMP1/IFNG/ICAM1/TLR2/CTLA4/IL1B/CCL2/JUN	1.85E-13
hsa04662	B cell receptor signaling pathway	AKT1/GRB2/RAF1/PIK3R1/IKBKB/MAPK1/NFKB1/PIK3CD/PIK3CA/CHUK/PIK3CG/JUN/MAP2K1	3.53E-12
hsa04080	Neuroactive ligand-receptor interaction	CNR2/FSHR/OPRK1/ADRB3/HTR1A/CHRM4/OPRM1/SSTR2/GLP1R/OPRL1/HTR4/TSHR/CHRM2/OPRD1/CNR1/ADRB2/PTGIR/CALCR/GCGR/ADRB1/AGTR2	7.86E-12
hsa04914	Progesterone-mediated oocyte maturation	AKT1/RAF1/PIK3R1/MAPK1/ADCY1/PIK3CD/PIK3CA/PIK3CG/GNAI1/ADCY5/MAP2K1/ADCY2	3.39E-10
hsa04370	VEGF signaling pathway	VEGFA/AKT1/RAF1/PIK3R1/MAPK1/SRC/PIK3CD/PIK3CA/PIK3CG/MAP2K1	5.22E-10
hsa04664	Fc epsilon RI signaling pathway	AKT1/GRB2/RAF1/PIK3R1/MAPK1/PIK3CD/PIK3CA/PRKCD/PIK3CG/MAP2K1/IL3	1.93E-09
