# Author Correction: Uncovering pharmacological mechanisms of Wu-tou decoction acting on rheumatoid arthritis through systems approaches: drug-target prediction, network analysis and experimental validation

**DOI:** 10.1038/s41598-018-34061-y

**Published:** 2018-10-30

**Authors:** Yanqiong Zhang, Ming Bai, Bo Zhang, Chunfang Liu, Qiuyan Guo, Yanqun Sun, Danhua Wang, Chao Wang, Yini Jiang, Na Lin, Shao Li

**Affiliations:** 1 Institute of Chinese Materia Medica, China Academy of Chinese Medical Sciences, Beijing, 100700 China; 2 MOE Key Laboratory of Bioinformatics and Bioinformatics Division, TNLIST, Department of Automation, Tsinghua University, Beijing, 100084 China; 3 Tianjin International Joint Academy of Biotechnology & Medicine, Tianjin, 300457 China

Correction to: *Scientific Reports* 10.1038/srep09463, published online 30 March 2015

This Article contains errors.

In Figure 1,

“Collagen-induced arthritis (CIA) mouse model”

should read:

“Collagen-induced arthritis (CIA) rat model”

In addition, in Figure 4A the panel showing the inflamed paw of the CIA group is incorrect.

Furthermore, the legend of Figure 4 contains an error where,

‘mice’

should read:

‘rats’

The correct Figure 4 and its accompanying legend appear below as Figure 1.

**Figure 1:**
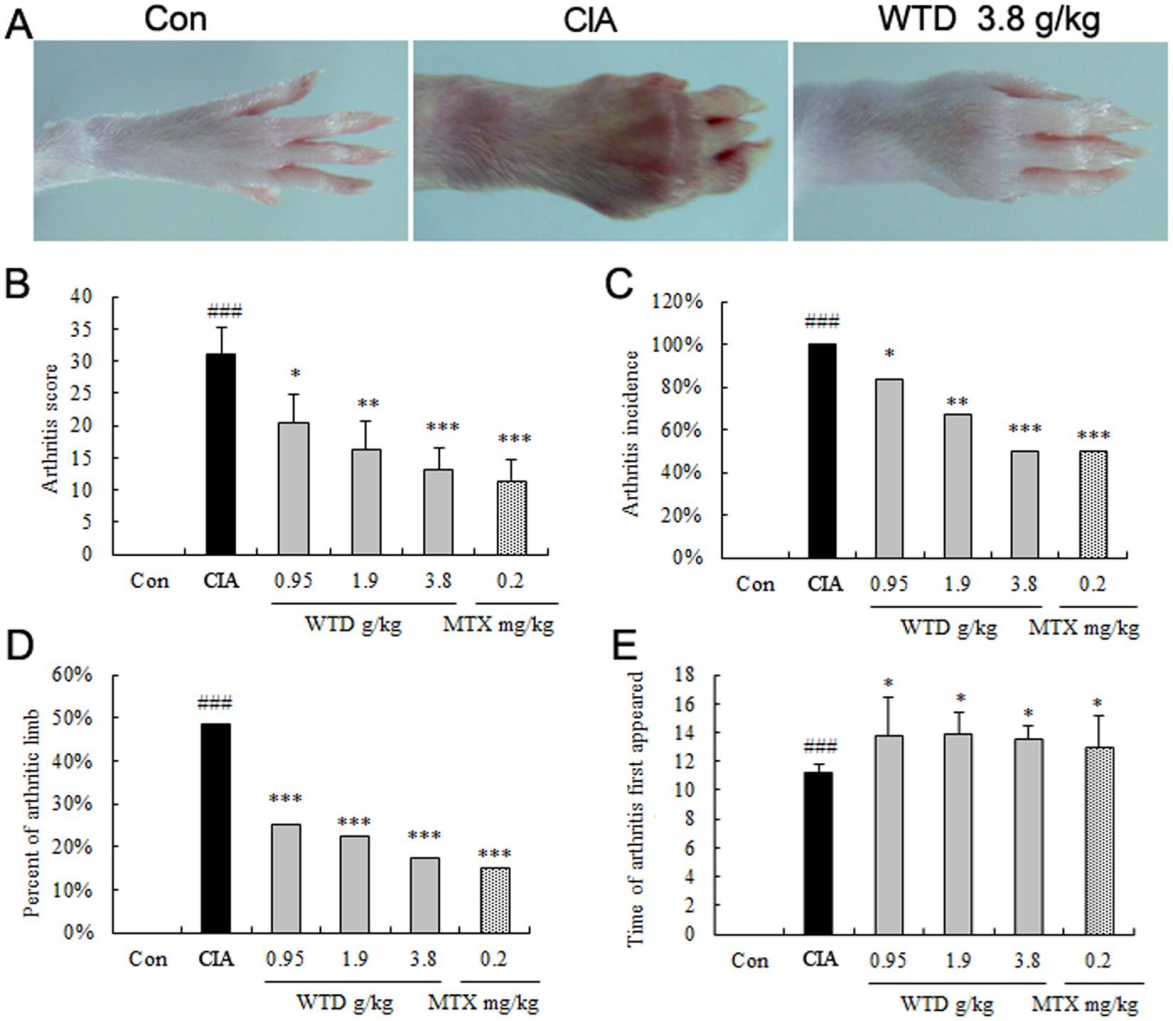
Effects of Wu-tou decoction (WTD) on severity of arthritis in collagen-induced arthritis (CIA) rats. (**A**) macroscopic evidence of arthritis such as erythema or swelling was markedly observed in vehicle-treated CIA rats, while dose of 3.8 g/(kg·day) WTD significantly attenuated arthritis severity in CIA rats; (**B**) Doses of 0.95~3.8 g/(kg·day) WTD significantly decreased the mean arthritis score in a dose-dependent manner compared with vehicle-treated CIA rats; (**C**) Doses of 0.95~3.8 g/(kg·day) WTD significantly decreased the arthritis incidence in a dose-dependent manner compared with vehicle-treated CIA rats; (**D**) Doses of 0.95~3.8 g/(kg·day) WTD significantly decreased the percentage of arthritis limbs in a dose-dependent manner compared with vehicle-treated CIA rats; (**E**) Doses of 0.95~3.8 g/(kg·day) WTD significantly increased the time of arthritis first appeared compared with vehicle-treated CIA rats. Data are represented as the mean±S.D. (n=12). ‘#’, P<0.05, comparison with the normal control. ‘*’, ‘**’, and ‘***’, P<0.05, P<0.01, and P<0.001, respectively, comparison with the vehicle control.

Finally, in the Methods section under the subheading ‘Treatment and groups’,

“The dosage selection for WTD [3.8 μg/(kg·day)] was nearly equivalent to RA patient dosage daily (42 g/person/day).”

should read:

“The dosage selection for WTD [3.8 g/(kg·day)] was nearly equivalent to RA patient dosage daily (42 g/person/day).”

